# Mass Spectrometry-Based Method to Study Inhibitor-Induced Metabolic Redirection in the Central Metabolism of Cancer Cells

**DOI:** 10.5702/massspectrometry.A0067

**Published:** 2018-06-14

**Authors:** Chie Araki, Nobuyuki Okahashi, Kousuke Maeda, Hiroshi Shimizu, Fumio Matsuda

**Affiliations:** 1 Department of Bioinformatic Engineering, Graduate School of Information Science and Technology, Osaka University

**Keywords:** ^13^C-metabolic flux analysis, isotope abundance, metabolic redirection, central metabolism, cancer cells

## Abstract

Cancer cells often respond to chemotherapeutic inhibitors by redirecting carbon flow in the central metabolism. To understand the metabolic redirections of inhibitor treatment on cancer cells, this study established a ^13^C-metabolic flux analysis (^13^C-MFA)-based method to evaluate metabolic redirection in MCF-7 breast cancer cells using mass spectrometry. A metabolic stationary state necessary for accurate ^13^C-MFA was confirmed during an 8–24 h window using low-dose treatments of various metabolic inhibitors. Further ^13^C-labeling experiments using [1-^13^C]glucose and [U-^13^C]glutamine, combined with gas chromatography-mass spectrometry (GC-MS) analysis of mass isotopomer distributions (MIDs), confirmed that an isotopic stationary state of intracellular metabolites was reached 24 h after treatment with paclitaxel (Taxol), an inhibitor of mitosis used for cancer treatment. Based on these metabolic and isotopic stationary states, metabolic flux distribution in the central metabolism of paclitaxel-treated MCF-7 cells was determined by ^13^C-MFA. Finally, estimations of the 95% confidence intervals showed that tricarboxylic acid cycle metabolic flux increased after paclitaxel treatment. Conversely, anaerobic glycolysis metabolic flux decreased, revealing metabolic redirections by paclitaxel inhibition. The gap between total regeneration and consumption of ATP in paclitaxel-treated cells was also found to be 1.2 times greater than controls, suggesting ATP demand was increased by paclitaxel treatment, likely due to increased microtubule polymerization. These data confirm that ^13^C-MFA can be used to investigate inhibitor-induced metabolic redirection in cancer cells. This will contribute to future pharmaceutical developments and understanding variable patient response to treatment.

## INTRODUCTION

Various metabolic inhibitors are used as medications or chemotherapeutic agents for a wide variety of conditions, including cancer.^1)^ Exposure to these inhibitors often affects cell viability by directly or indirectly affecting metabolic homeostasis.^2)^ In response to these changes, mammalian cells respond in-turn by redirecting carbon flow in the central metabolism. This supplies a required amount of raw materials and essential cofactors for adaptation to adverse conditions.^1,2)^ It is expected that a better understanding of cytotoxicity and the cellular mechanisms that underlie drug resistance can be achieved by analyzing the metabolic changes that occur after inhibitor exposure. To this end, flux analyses using stable isotope-labeled tracers have been developed and widely employed to investigate flux and metabolic redirections in cancer cells.^3,4)^ For example, cancer cells and tissues have been successfully cultured in medium containing a ^13^C-labeled carbon source that is subsequently incorporated into metabolites (^13^C-labeling experiments).^5)^ By measuring ^13^C labeling patterns in intracellular metabolites, or by examining mass isotopomer distributions (MIDs) using mass spectrometry, flux ratios at various metabolic branch points can be established.^6)^ These MID analyses have revealed various cancer-specific metabolic pathways that contribute to disease, such as reductive glutamine and C1 metabolism.^3,7–10)^

In addition to the MID analyses, ^13^C-metabolic flux analysis (^13^C-MFA) is a promising method to perform a more thorough investigation of inhibitor-induced metabolic redirection in central metabolism.^11,12)^ Using this tool, metabolic flux distributions in the central metabolism have been estimated from MIDs and carbon uptake and lactate production rates.^13–16)^ Despite several applications of ^13^C-MFA for mammalian cells^17–22)^ as well as preliminary analyses of the estradiol stimulated MCF-7 cells and verapamil treated HL-1 cardiomyocytes,^23)^ it remains unclear whether ^13^C-MFA could be applied to investigate the effects of metabolic inhibitors in cancer cells. It is because the ^13^C-MFA requires two stationary states.^24,25)^ Firstly, cells need to be maintained and examined at a metabolic stationary state during exponential growth. Secondly, the MIDs of intracellular metabolites must be measured and compared at an isotopically stationary state under which these MIDs have reached a plateau.^24,25)^ The modern ^13^C-MFA methodology strictly requires the two stationary states for statistically acceptable flux estimation with 95% confidence intervals (CIs) by a direct MID measurement of many intracellular metabolites. Thus, an analysis of dynamic metabolic events after inhibitor treatment is still challenging for ^13^C-MFA.

This study optimized a ^13^C-MFA method to evaluate metabolic redirection in cancer cells after treatment with various inhibitors used for cancer treatment. MCF-7 breast cancer cell line treated with the mitosis inhibitor paclitaxel (Taxol) was selected as a model for further investigation and model development. Paclitaxel is a chemotherapeutic agent used for many types of cancer, including breast carcinomas.^26)^ Further experiments using this model established that the metabolic and isotopic stationary states required for ^13^C-MFA could be achieved, occurring during within an 8–24 h window after low-dosage inhibitor treatment. Finally, flux redirection in the central metabolism of paclitaxel-treated MCF-7 cells was successfully evaluated by the developed method. This analysis indicates that ^13^C-MFA is indeed suitable for investigating inhibitor-induced metabolic redirection in cancer cells. This will contribute to developing future pharmaceuticals and understanding patient responses to treatment.

## MATERIALS AND METHODS

### MCF-7 culture

MCF-7 human breast carcinoma cells (3.6×10^5^ cells) were seeded in 5 mL Dulbecco’s modified Eagle’s medium (DMEM) containing 10% fetal bovine serum (FBS) and 1% penicillin/streptomycin (Wako) in 60-mm (diameter) plates and cultured for 15 h at 37°C with 5% CO_2_. For the ^13^C-labeling experiments, cells were cultured in 5 mL DMEM without glucose, L-glutamine, phenol red, sodium pyruvate, and sodium bicarbonate (Sigma-Aldrich, St. Louis, MO, USA), and supplemented with 20 mM [1-^13^C]glucose, 2 mM [U-^13^C] glutamine (Cambridge Isotope Laboratories, Andover, MA, USA, over 99% purity), 3.7 g/L sodium bicarbonate, and 10% dialyzed FBS (Life Technologies, Gaithersburg, MD, USA). Cultured medium (1 mL) was sampled for the analysis of extracellular metabolites. For ^13^C-labeling experiments, cells were cultured on 20 plates in parallel, and 10 plates were used for sampling of intracellular metabolites of cells cultured in DMEM containing [1-^13^C]glucose, and the other 10 plates containing [U-^13^C]glutamine at 9, 12, 16, 20, and 24 h from the start of ^13^C-labeling. The live cells were counted with trypan blue dye and a TC20 automated cell counter (BIO-RAD, Hercules, CA, USA). Trypsin was added to the plates and activated in 37°C for 1 min. After collecting the cells, the live cells were counted with TC20. For extracellular metabolite measurements, culture medium (1.0 mL) was collected, mixed with an equal volume of 20 mM pimelate solution (internal standard), and filtered through a filter cartridge (0.45-μm pore size). Extracellular metabolite concentrations were determined by the previously described methods.^25)^

### Metabolite extraction and derivatization

Intracellular metabolites were extracted using methanol/water/chloroform.^25)^ The medium was removed, and cells were rinsed with PBS. Next, 800 μL of −80°C methanol containing 5 μM ribitol (internal standard) was added to quench metabolism. Both solution and cells were collected *via* scraping. Cell lysates were transferred to fresh sample tubes, and 800 μL of cold chloroform and 320 μL of cold water were added. After vortexing and centrifugation, the top aqueous layer was collected and dried under airflow. Dried metabolites were dissolved in 50 μL of 40 mg/mL methoxyamine hydrochloride in pyridine and held at 30°C for 90 min. Finally, 50 μL of MTBSTFA containing 1% TBDMCS was added and held at 60°C for 30 min.

### Gas chromatography/mass spectrometry analysis

Gas chromatography/mass spectrometry (GC/MS) analysis was performed using an Agilent 7890 GC with DB-5MS capillary column (Agilent Technologies) connected to an Agilent 5975 MSD. The GC/MS was operated under electron impact (EI) ionization at 70 electron volts (eV). In splitless mode, a 1 μL sample was injected at 250°C, using helium as the carrier gas at a flow rate of 1 mL/min. For the analysis of central metabolites derivatives, the GC oven temperature was held at 70°C, increased to 280°C at a rate of 3°C/min for a total run time of approximately 75 min. The MS source and quadrupole were held at 230°C and 150°C, respectively, and the detector was operated in the selected ion monitoring mode. The *m*/*z* values for the ^13^C-labeling of each metabolite are shown in the Supplementary Data S1.

### ^13^C-Metabolic flux analysis

MFA was performed using a metabolic model of *Homo sapiens* similar to that used in the previous studies.^17–21)^ The model includes 85 reactions and 81 metabolites in the pathways for glycolysis, pentose phosphate, TCA cycle, anaplerosis, and lipid biosynthesis, as well as the metabolic branch from 3-phosphoglyceric acid (PGA) to serine biosynthesis and C1 pathway (Supplementary Data S1). In order to avoid rank deficiency during the calculation, metabolic flux levels of the output reactions for Asn and lactate were fitted to the measured values by calculating the RSS values. The metabolic flux levels of the two output reactions were estimated by the ^13^C-MFA, since the metabolic flux levels toward the C1 metabolism (PGA→C1 metabolism) and fatty acid biosynthesis (AcCoA_c→fatty acid biosynthesis) were not experimentally determined in this study. The metabolic branch was included because it has been reported that a significant amount of PGA is supplied to the C1 metabolic pathway and is degraded into CO_2_, regenerating NADPH.^8)^ In addition, intracellular compartmentalization between the cytosol and mitochondria was ignored for pyruvate, citrate, and α-ketoglutarate (αKG) as introducing intracellular compartmentalization failed to improve model fitting (data not shown), since, similar simplification has also been performed in previous ^13^C-MFA studies.^17,19,21,27)^ The metabolic flux levels of the other input and output reactions were fixed to the observed values, and not used for the residual sum of squares (RSS) calculation. Fluxes for biomass of MCF-7 were calculated from the precursor and dry cell weight data.^28)^

All data analysis for the ^13^C-MFA experiments were performed using a Python version of OpenMebius^29)^ implemented in Python 2.7.9 with NumPy 1.9.1, SciPy 0.4.2, PyOpt 1.2, and parallel Python 1.6.4 modules. In this study, ^13^C-labeling patterns of [M−85]^+^ of Pyr; [M−85]^+^ of Mal; [M−85]^+^ of Cit; [M−85]^+^ and [M−57]^+^ of Ala; and [f302]^+^, [M−57]^+^, and [M−85]^+^ of Asp obtained from the cells labeled by [U-^13^C]glutamine were used for the ^13^C-MFA. Here, [f302]^+^ indicated a fragment ion (*m*/*z* 302) commonly observed in the EI mass spectra of TBDMS derivatized amino acids. Furthermore, ^13^C-labeling patterns of [M−85]^+^ of αKG; [M−85]^+^ of Mal; [M−85]^+^ of 3PG; [M−85]^+^; and [M−57]^+^ of Ala; and [f302]^+^, [M−57] ^+^, and [M−85]^+^ of Asp obtained from the cells labeled by [1-^13^C]glucose were used for the ^13^C-MFA. The effect of naturally occurring isotopes was removed from the raw mass spectrometry data. Metabolic flux levels were estimated by minimizing the RSS between experimentally measured and simulated MIDs using the sequential least squares programming (SLSQP) function implemented in PyOpt 1.2^30)^: 
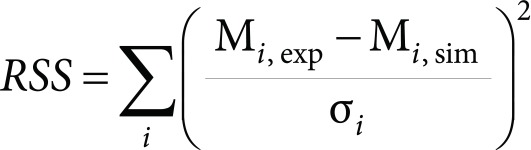
 where M*_i_*_, exp_ and M*_i_*_, sim_ represent the experimentally measured and simulated MID values of the *i*-th metabolites, respectively, and σ represents the standard deviation (0.015). Finally, 95% CIs were estimated by the grid search method.^30)^ The ATP regeneration rate by OxPHOS was calculated using a P/O ratio of 2.3.^31)^

## RESULTS

### The effects of inhibitor treatment on culture profiles and material balance in MCF-7 cells

**Figure 1:**
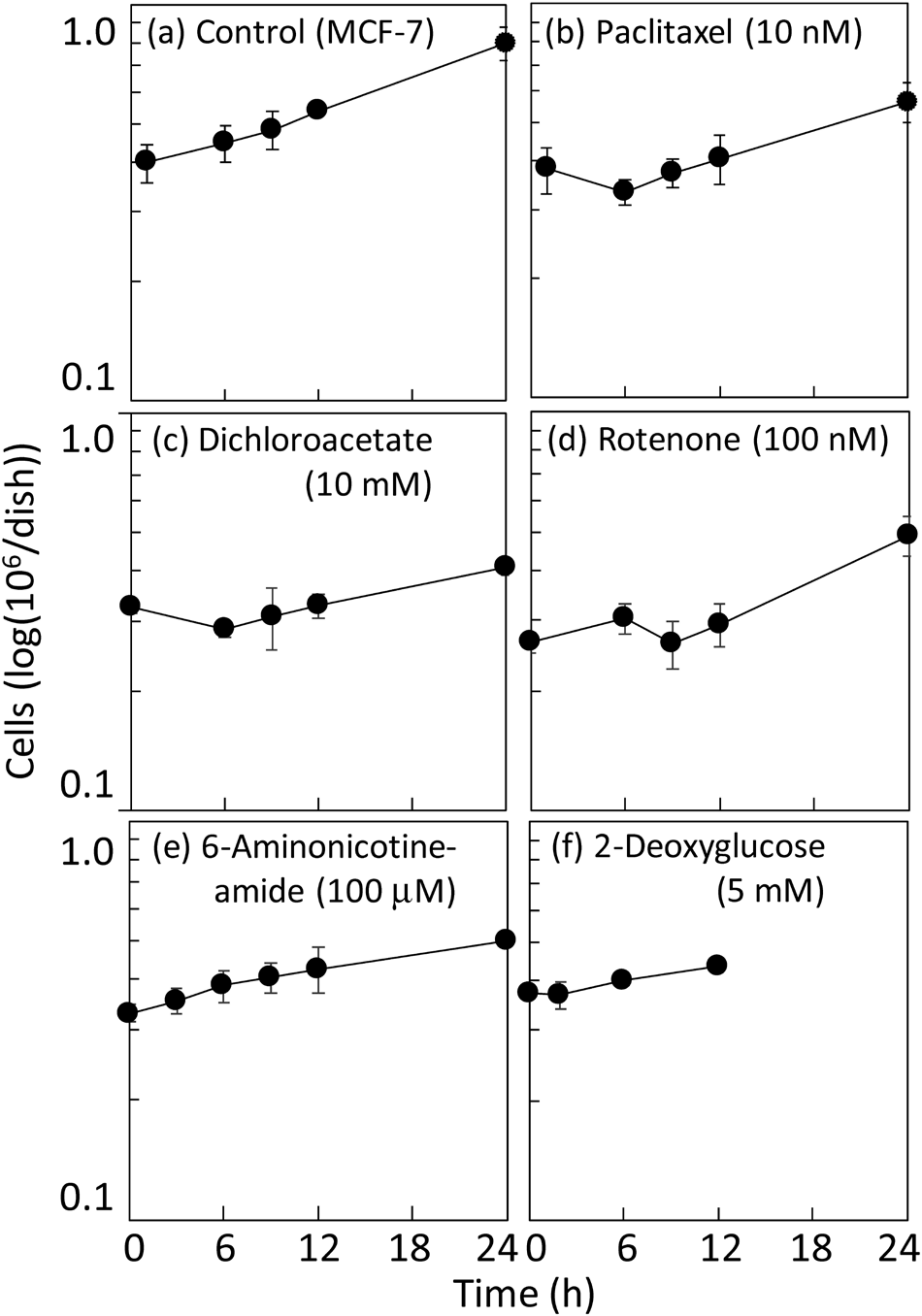
Fig. 1. Growth curves of MCF-7 cells that were untreated (a) or treated with 10 nM paclitaxel (b), 10 mM dichloroacetate (c), 100 nM rotenone (d), 100 μM 6-aminonicotineamide (e), or 5 mM 2-deoxyglucose (f) for 15 h before media was replaced with inhibitor-free media.

In order to identify the exponential growth of MCF-7 human breast carcinoma cells, culture profiles and growth curves were first investigated (Fig. 1a). This analysis indicated that MCF-7 cells were under exponential growth from 6–24 h after initial seeding. Suitable concentrations for subsequent inhibitor treatment analysis were then evaluated using dose–response analyses. In particular, the number of live MCF-7 cells numbers at 24 h after paclitaxel treatment showed that growth inhibition occurred in a dose-dependent manner (data not shown). As cells maintained in an exponential growth phase are essential for the ^13^C-labeling experiment, a paclitaxel concentration that elicited 50% inhibition (10 nM) was employed for subsequent study.

Effect of paclitaxel treatment on the culture profile was determined by the addition of paclitaxel. A growth curve analysis revealed that MCF-7 cell numbers decreased from 0–6 h after treatment (Fig. 1b). This acute response is supported by previous studies indicating increased cell apoptosis at these concentrations of paclitaxel.^32)^ After this initial period of cell death, the remaining cells then increased in number in a time-dependent manner. These results indicate that exponential growth (or a metabolic stationary phase) occurred from 6–24 h (Fig. 1b). Furthermore, the recovery in growth suggests that MCF-7 cells have adapted to paclitaxel treatment and we hypothesized that this was likely due to changes in metabolic flux distribution.

In addition to paclitaxel, similar analyses were performed for several other metabolic inhibitors, including dichloroacetate (an inhibitor of the pyruvate dehydrogenase kinase), rotenone (a mitochondrial complex I inhibitor), 6-aminonicotinamide (an inhibitor of NADP^+^-dependent 6-phosphogluconate dehydrogenase), and 2-deoxyglucose (glycolytic inhibitor) (Figs. 1c–1f). The culture profiles of each of these inhibitors were similar to paclitaxel-treated cells, reaching an exponential growth phase between 8–24 h. These results demonstrate that the exponential growth (or pseudo-metabolic stationary) state required for accurate ^13^C-MFA can likely be attained for many inhibitors by selecting a dose that leads to partial inhibition of cell growth. However, for this study, paclitaxel was selected as the model compound for further development.

**Figure 2:**
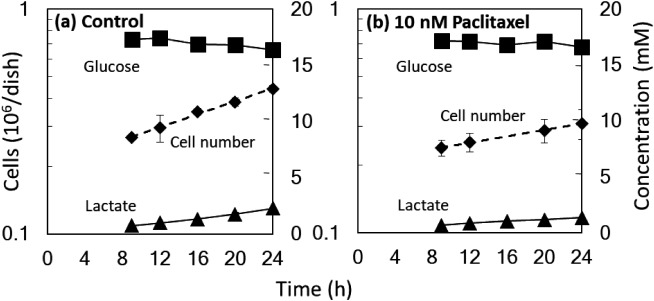
Fig. 2. Culture profiles of paclitaxel-treated MCF-7 cells. The concentrations of various media components are also indicated, including glucose and lactate. Specific rates were determined from data shown in Table 1.

In order to obtain the material balance data required for ^13^C-MFA, cell culture was repeated to determine the specific growth rates of MCF-7 cells during the identified metabolic stationary phase (9–24 h) (Fig. 2). The specific growth rates were determined to be 0.033 h^−1^ for control cells (Fig. 2a) and 0.014 h^−1^ for paclitaxel-treated cells (approximately 43% of the control; Fig. 2b). Further analysis of the culture medium showed that the concentrations of extracellular glucose and lactate had either decreased or increased in a time-dependent manner, respectively (Fig. 2a). The specific rates for glucose consumption and lactate production in control cells were determined from the time course data to be 858±351 and 1451±26 nmol (10^6^ cells)^−1^ h^−1^, respectively (Table 1). These data suggested that paclitaxel treatment affected the material balance of MCF-7 cells due to the fact that the specific rates of glucose consumption (673±164 nmol [10^6^ cells]^−1^ h^−1^) and lactate production (821±53 nmol [10^6^ cells]^−1^ h^−1^) were 78 and 56% of the control rates, respectively (Table 1). The consumption and production rates for amino acids were also determined from the medium component analysis (Table 1).

**Table 1:** Table 1. Specific production and consumption rates.

	Specific rate (nmol (10^6^ cells)^−1^ h^−1^)^1)^	
	Control	10 nM Paclitaxel
Consumption		
Glucose	858±351	673±164
Glutamine	91.2±25.6	95.3±11.0
Arginine	46.5±2.7	49.3±9.6
Production		
Lactate	1451±26	821±53
Glutamate	22.5±0.8	34.9±2.2
Proline	3.7±0.2	5.3±1.4
Aspartate	4.2±1.2	9.8±0.3
Asparagine	10.5±0.6	15.2±3.6
Alanine	25.6±1.2	23.1±1.3

^1)^All data are mean±standard deviations of three independent cultures.

### Confirmation of an isotopic stationary state

**Figure 3:**
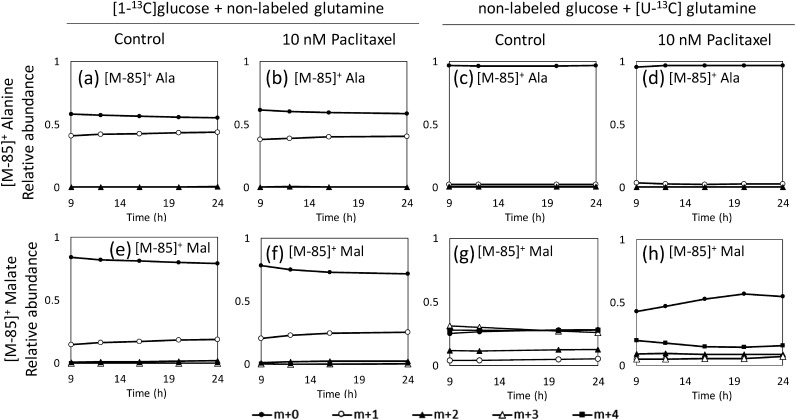
Fig. 3. The ^13^C-labeling kinetics of intracellular metabolites. Control (a, c, e, and g) and paclitaxel-treated (b, d, f, and h) MCF-7 cells were iteratively collected at 9, 12, 16, 20, and 24 h after labeling with [1-^13^C]glucose (a, b, e, and f) or [U-^13^C]glutamine (c, d, g, and h). Free metabolites were extracted and mass isotopomer distributions (MIDs) determined by gas chromatography-mass spectrometry. Any effects derived from naturally occurring stable isotopes were corrected for [M−85]^+^Ala: the fragment of alanine including carbons 2–3 (a–d), [M−85]^+^ Mal: the fragment of malate including carbons 2–3–4 (e–f).

Next, an isotopic stationary state was confirmed using a ^13^C-labeling experiment. To achieve this, cell media were exchanged with DMEM containing either [1-^13^C]glucose or [U-^13^C]glutamine at the 0 h timepoint. Intracellular free metabolites were extracted and ^13^C-labeling determined by gas chromatography-mass spectrometry following derivatization with *tert*-butyldimethylsilyl (TBDMS) ethers (Supplementary Fig. S1). Figures 3a–3d show time-course data summarizing the mass isotopomer distributions (MIDs) of the [M−85]^+^ fragment of alanine (Ala). The relative abundances of the m+0 (non ^13^C-labeled) and m+1 (single ^13^C-labeled) forms of Ala were both close to a maximal plateau for all samples 24 h after labeling (Figs. 3a–3d). The MIDs of the [M−85]^+^ fragment of malate (Mal) were also near a plateau after 24 h (Figs. 3e–3h). Similar results were observed for each of the measured metabolites (Supplementary Fig. S2). Although more comprehensive ^13^C-labeling would have provided accurate flux analysis, a longer cell cultivation time was determined to not be feasible under the current experimental conditions because an exponential cell growth phase could not be maintained. This was due to cell confluency being reached at 30–36 h (data not shown). Therefore, only MID data 24 h after labeling were employed for flux estimation since an isotopically stationary state was almost attained.

Subsequent MID data showed that ^13^C-labeling of metabolites in MCF-7 cells was affected by paclitaxel treatment. Specifically, for cells cultured in [1-^13^C]glucose, the relative abundances of the m+1 signal of [M−85]^+^Ala were 0.440 (Fig. 3a) and 0.405 (Fig. 3b) at 24 h in control and paclitaxel-treated cells, respectively. For [1-^13^C]glucose, equal amounts of unlabeled and [3-^13^C]-labeled Ala were produced *via* the Embden–Meyerhof–Parnas (EMP) pathway. Conversely, only unlabeled Ala molecules were produced *via* the oxidative pentose phosphate (oxPP) pathway as the ^13^C atom was discarded. Thus, the branching ratios of metabolic flux between the oxPP and EMP pathways (oxPP/EMP) could be estimated from the labeling patterns of Ala.^12)^ If this branching ratio increases, metabolic change would be observed as a decrease in the relative intensities of mass signals derived from the m+1 signal of [M−85]^+^Ala. These data suggest that paclitaxel treatment increases the oxPP/EMP branching ratios of MCF-7 cells (Figs. 3a and 3b).

### Estimation of metabolic flux distribution using ^13^C-MFA

As both metabolic and isotopic stationary states were established, it was confirmed that ^13^C-MFA could be applied to examine the effects of paclitaxel treatment in MCF-7 cells (Figs. 1 and 3). In addition to stationary states, ^13^C-MFA also requires a direct MID measurement of many intracellular metabolites. For the purpose, a parallel labeling method was employed to increase MID measurement for a better estimation of metabolic flux using an experimental design shown in Supplementary Fig. S1.^27,33–35)^ In this case, MCF-7 cells were cultured in parallel with media containing (a) [1-^13^C]glucose and unlabeled glutamine or (b) unlabeled glucose and [U-^13^C]glutamine. After 24 h, two sets of MID data were obtained from these MCF-7 cells (Supplementary Data S1).

**Figure 4:**
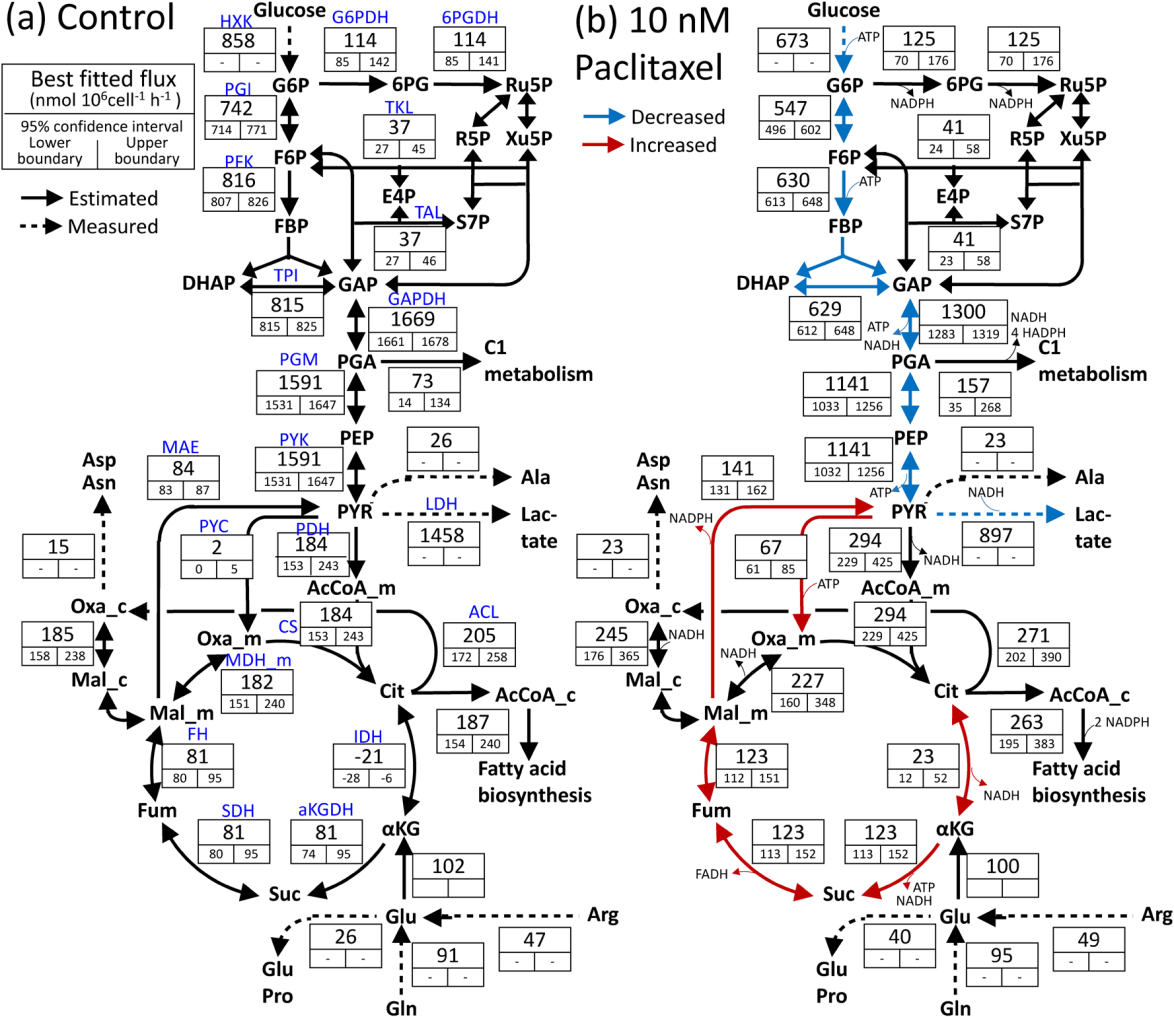
Fig. 4. Metabolic flux distribution in control (a) and MCF-7 cells treated with 10 nM paclitaxel (b). The flux levels used in this study are shown in the metabolic model. Each panel with numbers represents a best-fit estimation of metabolic flux with lower and upper 95% confidence intervals (CIs). Dotted arrows represent reactions where metabolic flux levels were determined from a medium component analysis (Table 1). The red and blue arrows in panel (b) show reactions where metabolic flux levels were either increased or decreased by paclitaxel treatment. Significance was indicated by non-overlapping 95% CIs between any two conditions. The blue labels indicate reaction names. All abbreviations are defined in Supplementary Data S3. Cofactors in the reactions are shown in panel (b).

Next, the metabolic distributions of control and paclitaxel-treated MCF-7 cells were determined from material balances 24 h after labeling (Table 1) and from MID data (Fig. 4 and Supplementary Data S1). The metabolic model used in this study was based on prior ^13^C-MFA studies using cancer cells^21,27)^ and included glycolysis, the pentose phosphate pathway, the TCA cycle, anaplerotic reactions, fatty acid biosynthesis, and the metabolic branch from 3-phopshoglycerate (PGA) to the C1 metabolic pathway (PGA→C1 metabolism; Fig. 4 and Supplementary Data S1). As the studied system was over-determined, goodness of fit could be validated by χ^2^ tests.^36)^ Although the threshold residual sum of squares (RSS) was 47.4, the RSS of control and paclitaxel-treated cells were 36.9 and 42.1, respectively (Supplementary Data S2).

This ^13^C-MFA highlighted various facets of the cancer-specific metabolism of MCF-7 cells, such as an active anaerobic glycolysis pathway due to the Warburg effect.^37–39)^ In control MCF-7 cells, the metabolic flux of the glyceraldehyde-3-phosphate dehydrogenase (GAPDH) reaction in glycolysis was 1,669 nmol (10^6^ cells)^−1^ h^−1^. This was 10-fold greater than that of the TCA cycle and the oxidative pentose phosphate pathway. In addition, a large amount of α-ketoglutaric acid (αKG; 102 nmol [10^6^ cells]^−1^ h^−1^) was also supplied from glutamine (Gln) and arginine (Arg). This was 11.8% of the glucose uptake rate and αKG was primarily catabolized *via* oxidative TCA reactions and malic enzyme (malate dehydrogenase) to produce lactate.

### Metabolic redirection after paclitaxel treatment

Although it has been shown that paclitaxel treatment inhibits tubulin polymerization, blocks cell replication, arrests cells in G2/M, and induces apoptosis,^32)^ the effects on central metabolism are unclear. This is because metabolic profiling of paclitaxel treatment using immortalized human pancreatic cell lines showed no significant changes in central metabolism.^40)^ However, a comparison of estimated metabolic flux distributions in this study clearly indicated that paclitaxel treatment reduced metabolic flux into the EMP pathway that converts glucose into lactate for anaerobic glycolysis (blue arrows in Fig. 4b). For example, metabolic flux into the entry reaction of the EMP pathway (PGI) in control cells was 742 nmol (10^6^ cells)^−1^ h^−1^. These levels decreased by 27% in paclitaxel-treated cells (547 nmol [10^6^ cells]^−1^ h^−1^). However, the metabolic flux levels of the oxPP pathway in control and paclitaxel-treated cells were similar to each other (114 and 125 nmol [10^6^ cells]^−1^ h^−1^, respectively). This indicates that the branching ratio of oxPP/EMP had increased from 0.15 to 0.23 after paclitaxel treatment. This observed metabolic redirection at the EMP/oxPP branch point is consistent with the previously observed MID shifts in [M−85]^+^Ala (Figs. 3a and 3b).

The paclitaxel-induced increase in metabolic flux was observed in reactions found in the lower half of the TCA cycle and two reactions in the anaplerotic pathway, malate dehydrogenase (MAE) and pyruvate carboxylase (PYC) (red arrows in Fig. 4b). For the case of the αKG dehydrogenase (αKGDH) reaction, the metabolic flux level in the paclitaxel-treated sample (123 nmol [10^6^ cells]^−1^ h^−1^) was 1.5-fold higher than that of the control sample (81 nmol [10^6^ cells]^−1^ h^−1^). As the αKG supply from Gln and Arg in the paclitaxel-treated sample (102 nmol [10^6^ cells]^−1^ h^−1^) was similar to that of the control cells (100 nmol [10^6^ cells]^−1^ h^−1^), this increase in αKGDH flux was derived from the redirection of metabolic flux in the isocitrate dehydrogenase (IDH) reaction. Indeed, the metabolic flux of the IDH reaction was altered from being reversed (reductive, −21 nmol [10^6^ cells]^−1^ h^−1^) to forward (oxidative, 23 nmol [10^6^ cells]^−1^ h^−1^) in the paclitaxel-treated sample (Fig. 4).

### Cofactor balance

**Figure 5:**
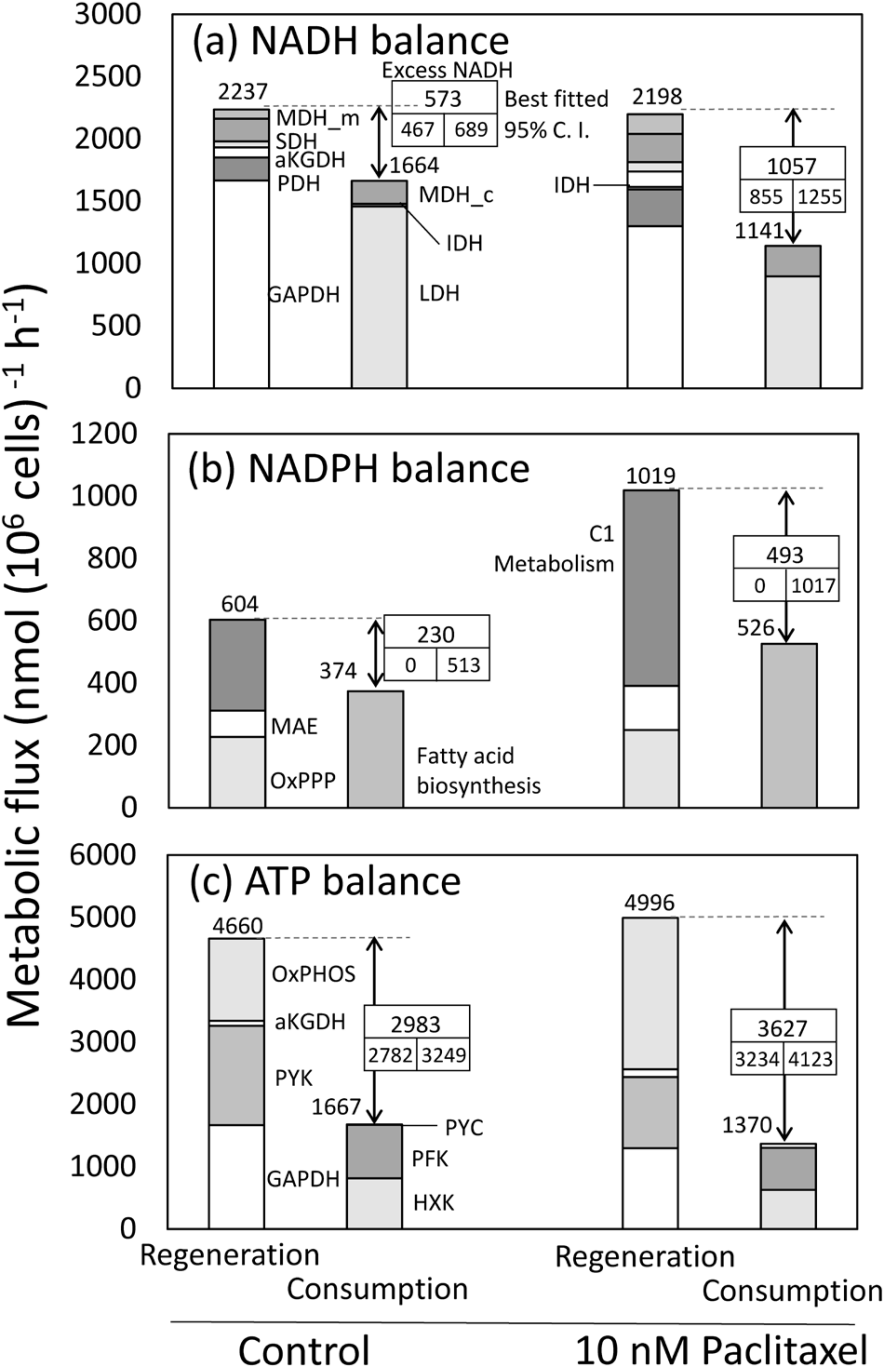
Fig. 5. Cofactor regeneration and consumption in the central metabolic network. The specific rates for regeneration and consumption of NADH (a), NADPH (b), and ATP (c) were obtained from best-fit metabolic flux distributions (Fig. 4). Gaps between regeneration and consumption rates, with their 95% confidence intervals (CIs), are shown. It was hypothesized that excess NADH was used for ATP regeneration in the oxidative phosphorylation (OxPHOS) pathway with a P/O ratio of 2.3.

Finally, the metabolic reactions responsible for the regeneration and consumption of various cofactors (ATP, NADH, and NADPH) were characterized in the central metabolic network (Fig. 4b). This allowed an investigation of a balance between cofactor regeneration and consumption rates. Figure 5a summarizes the NADH balance in control and paclitaxel-treated cells. For control cells, the NADH regeneration rate was determined to be 2,237 nmol (10^6^ cells)^−1^ h^−1^ (Fig. 5a), with 75 and 22% of NADH regenerated by the GAPDH reaction of glycolysis and reactions in the TCA cycle, respectively. On the other hand, the NADH consumption explained in the central metabolic network was 1,664 nmol (10^6^ cells)^−1^ h^−1^ (Fig. 5a). The primary consumer of NADH was found to be the lactate dehydrogenase (LDH) reaction, consuming 87% of the NADH regenerated by GAPDH. The gap between total NADH regeneration and consumption was determined to be 573 nmol (10^6^ cells)^−1^ h^−1^ and the 95% CI for this gap was calculated to be 467–689 nmol (10^6^ cells)^−1^ h^−1^ (Supplementary Data S1, 95% CI of r107_OxPHOS). The gap indicates that excess NADH was consumed as an electron donor in the electron transport system of oxidative phosphorylation (OxPHOS).

For paclitaxel-treated MCF-7 cells, the rate for excess NADH regeneration was determined to be 1,057 nmol (10^6^ cells)^−1^ h^−1^ with a 95% CI of 855–1255 nmol (10^6^ cells)^−1^ h^−1^. This is approximately 1.8-fold higher than control cells (Fig. 5a). This increase was ascribed to decreased NADH consumption by LDH and increased NADH regeneration by the TCA cycle (Fig. 5a). For NADPH, the estimated NADPH balance was deemed unreliable due to a large 95% CI of C1 metabolism and fatty acid biosynthesis flux (Fig. 5b).

ATP balance was also estimated from the OxPHOS rate calculated from NADH balance and the metabolic flux of reactions responsible for ATP regeneration and consumption. Figure 5c shows that the ATP regeneration rate in control cells was 4,660 nmol (10^6^ cells)^−1^ h^−1^ and the ratio between ATP regeneration by substrate phosphorylation (GAPDH and pyruvate kinase [PYK]) and by OxPHOS was established as 70 : 28. Of this regenerated ATP, 36% was consumed during the phosphorylation of glucose by hexokinase (HXK) and phosphofructokinase (PFK) during glycolysis. The gap between total regeneration and consumption was 2,983 nmol (10^6^ cells)^−1^ h^−1^, with a 95% CI of 2,782–3,249 nmol (10^6^ cells)^−1^ h^−1^ (Supplementary Data S1, 95% CI of r108_ATPx). These results suggest that ATP is being used for various cell functions, including cell component biosynthesis and cell division. The ATP regeneration rate in paclitaxel-treated cells was 4,996 nmol (10^6^ cells)^−1^ h^−1^. This was similar to levels found in control cells (Fig. 5c). However, the contribution ratio between substrate level phosphorylation and OxPHOS was 49 : 49, suggesting that ATP regeneration was more dependent on OxPHOS and the mitochondrial electron transport system. As ATP consumption during glycolysis was reduced, the regeneration rate of excess ATP was elevated to 3627 nmol (10^6^ cells)^−1^ h^−1^, with a 95% CI of 3,234–4,123 nmol (10^6^ cells)^−1^ h^−1^ (Fig. 5c).

## DISCUSSION

This study has demonstrated that ^13^C-MFA can be used to investigate metabolic redirection in inhibitor-treated cancer cells using paclitaxel-treated MCF-7 cells as a model. Since both metabolic and isotopic stationary states are required to apply ^13^C-MFA,^24,25)^ an important outcome of this study is therefore a confirmation that MCF-7 cells reach two stationary states after 10 nM paclitaxel treatment (Figs. 1 and 3). In particular, it was shown that MCF-7 cells were in a metabolic stationary state 8–24 h after paclitaxel treatment as an exponential growth phase was observed. Further measurements of intracellular metabolites by mass spectrometry revealed that MIDs had reached a plateau within this period, indicating an isotopically stationary state. Using these two stationary states, the overall distribution of flux in the central metabolism of untreated and paclitaxel-treated MCF-7 cells was successfully determined by ^13^C-MFA of an over-determined metabolic system by direct MID measurement of intracellular metabolites in the parallel labeling experiment, the goodness-of-fit analysis by the χ^2^ statistics, and the estimation of 95% CIs (Figs. 3 and 4).^34,36,41)^

Using this newly established ^13^C-MFA system, various aspects of the metabolism in MCF-7 cells and response to inhibitor treatment were revealed. A cancer-specific metabolism such as the Warburg effect was clearly observed in control cells since the metabolic flux of glycolysis was 10-fold larger than the TCA cycle (Fig. 4). In addition, glutamine was largely catabolized *via* oxidative TCA reactions and malate dehydrogenase to produce lactate. These trends were similar to a recent study using ^13^C-MFA to investigate lung cancer cells (A549)^27)^ and B-cells.^21)^ However, the result is inconsistent with another ^13^C-MFA study examining MCF-7 cells, probably because of differences in culture conditions and experimental design.^23)^ The result of this study also revealed that some αKG was converted into citrate (Cit) by the reversed reaction of isocitrate dehydrogenase (IDH) (Fig. 4). The reductive glutamine metabolism has been observed in various cancer cells and tissues and is therefore likely a marker of cancer-specific metabolism.^3,7)^ In addition, the ^13^C-MFA suggested that MCF-7 cells adopted a paclitaxel-treated condition by reducing their dependency on cancer-specific metabolism. Moreover, metabolic flux levels of other cancer-specific metabolic pathways such as MAE and PYC were upregulated by the paclitaxel treatment suggesting roles in the cancer metabolism.^42–44)^ A purpose of the metabolic redirection could be estimated from the quantitative determination of metabolic flux levels. The cofactor balance data (Fig. 5) indicated that excess ATP regeneration was elevated in paclitaxel-treated MCF-7 cells. The results suggested that the metabolic redirection is for the activation of ATP dependent microtubulin polymerization and for the ATP dependent excretion of paclitaxel.^32)^

However, the culture profiles and ^13^C-labeling kinetic data (Figs. 1 and 3) suggest some limitations in experimental design. The first limitation is that ^13^C-MFA cannot presently be applied to the study of organs as the method requires detailed material balance data, including carbon uptake and lactate production rates (Table 1). This currently limits the technique to cells or tissues cultured in media. A second limitation is that the inhibitor dose range is quite limited as a relatively prolonged metabolic stationary state is required after treatment to perform ^13^C-MFA. Therefore high-dose treatments that induce acute cell death would be unsuitable. Thirdly, the time points used for the analysis were limited since the metabolic state of MCF-7 cells changed in a time-dependent manner after 10 nM paclitaxel treatment (Fig. 1b). While, paclitaxel-induced cell death was observed between 6–9 h (Fig. 1b), the remaining cells entered an exponential growth phase after the cell death phase, indicating that MCF-7 cells may have adapted to paclitaxel treatment. This allowed metabolic flux distribution after the metabolic adaptation to be evaluated by ^13^C-MFA. A similar adaptation and exponential growth phase was observed for other metabolic inhibitors during the 8–24 h period, suggesting that ^13^C-MFA could be applied to analyze metabolic redirection induced by other inhibitors. A final limitation was the incomplete isotopically stationary states measured during the study (Fig. 3). Since longer cultivation times for more complete isotopically stationary states could conflict with the exponential growth required to establish a metabolic stationary state, culture conditions should be optimized for each unique experiment using different inhibitors and cell lines. If isotopically stationary states cannot be assumed, dynamic or isotopically non-stationary (INST)-MFAs using ^13^C-labeling time-course data have been developed that could be applied for mammalian cell analysis.^19,21,45,46)^ The INST-MFA requires additional data concerning the intracellular concentration of all intermediates in the metabolic network, as well as more computational time to estimate metabolic flux distributions.

In summary, this study demonstrate that ^13^C-MFA can be successfully applied to investigate metabolic redirection in the cancer cells due to inhibitor treatments. Using the validated metabolic and isotopic stationary states, ^13^C-MFA was used to evaluate redirection of central metabolism in paclitaxel-treated MCF-7 cells, revealing the importance of metabolic flux into the TCA cycle and anaerobic glycolysis in the adaptation process. Although this study has provided validation of the ^13^C-MFA method, more acute metabolic responses (<12 h) should be investigated for a more detailed understanding of the metabolic adaptation process. This would reveal where the metabolic state was transiently changed during the early response to inhibitor treatment. In addition, any regulatory mechanisms responsible for metabolic redirection cannot be estimated from the metabolic flux data alone.^12,47,48)^ Therefore, a more comprehensive analysis of dynamic metabolic adaptation mechanisms due to drug treatment could be revealed using ^13^C-based analysis in combination with other metabolic data. The present study suggests that ^13^C-MFA is suitable for investigating inhibitor-induced metabolic redirection in cancer cells. This will aid the development of better targeted pharmaceuticals and understanding different patient responses to treatment.^49)^

## Notes

Mass Spectrom (Tokyo) 2018; 7(1): A0067

## Notes

Mass Spectrometry Society of Japan bestowed the MSSJ Research Award on Fumio Matsuda. This is an original article related to the achievement.
