# Supplementary material for: Mass Spectrometry-Based Method to Study Inhibitor-Induced Metabolic Redirection in the Central Metabolism of Cancer Cells
Source: Mass Spectrom (Tokyo). 2018 Jun 14;7(1):A0067. doi: 10.5702/massspectrometry.A0067 (PMC6002601; doi:10.5702/massspectrometry.A0067)
Supplement: Supplementary Figures S1 and S2 [file massspectrometry-7-1-A0067-s001.pdf]

Figure S1. Design of the ^{13}C-labeling experiment

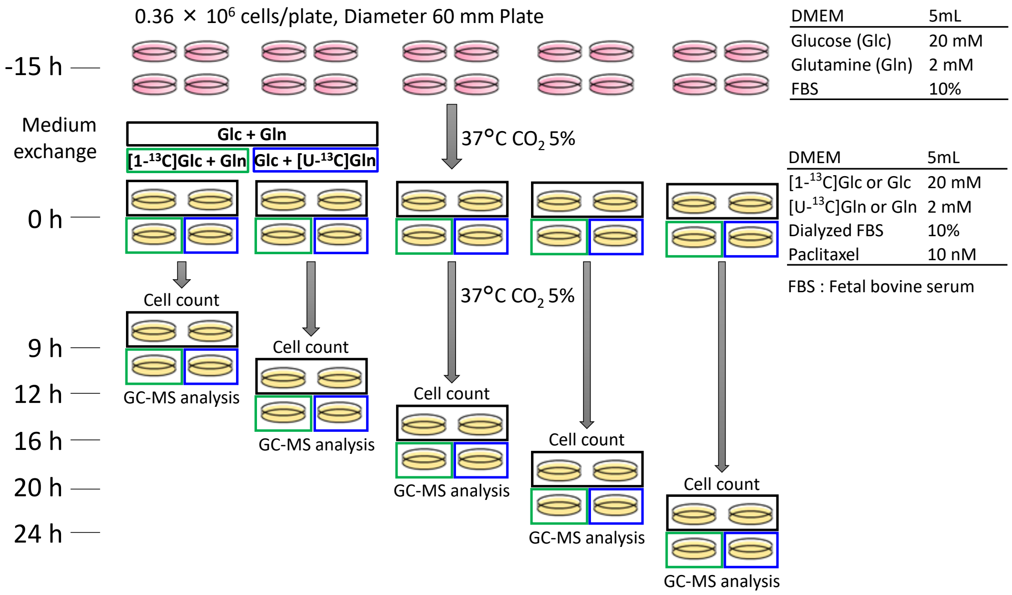

Figure S2. The ^{13}C-labeling kinetics of intracellular metabolites. Control (a and c) and paclitaxel-treated (b and d) MCF-7 cells were iteratively collected at 9, 12, 16, 20, 24 h after the labeling with [1-^{13}C]glucose (a and b) and [U-^{13}C]glutamine (c and d). Free metabolites were extracted and the mass isotopomer distributions (MIDs) were determined by gas chromatography–mass spectrometry. Effects derived from the naturally occurring stable isotopes were corrected for the precise determination of MID.

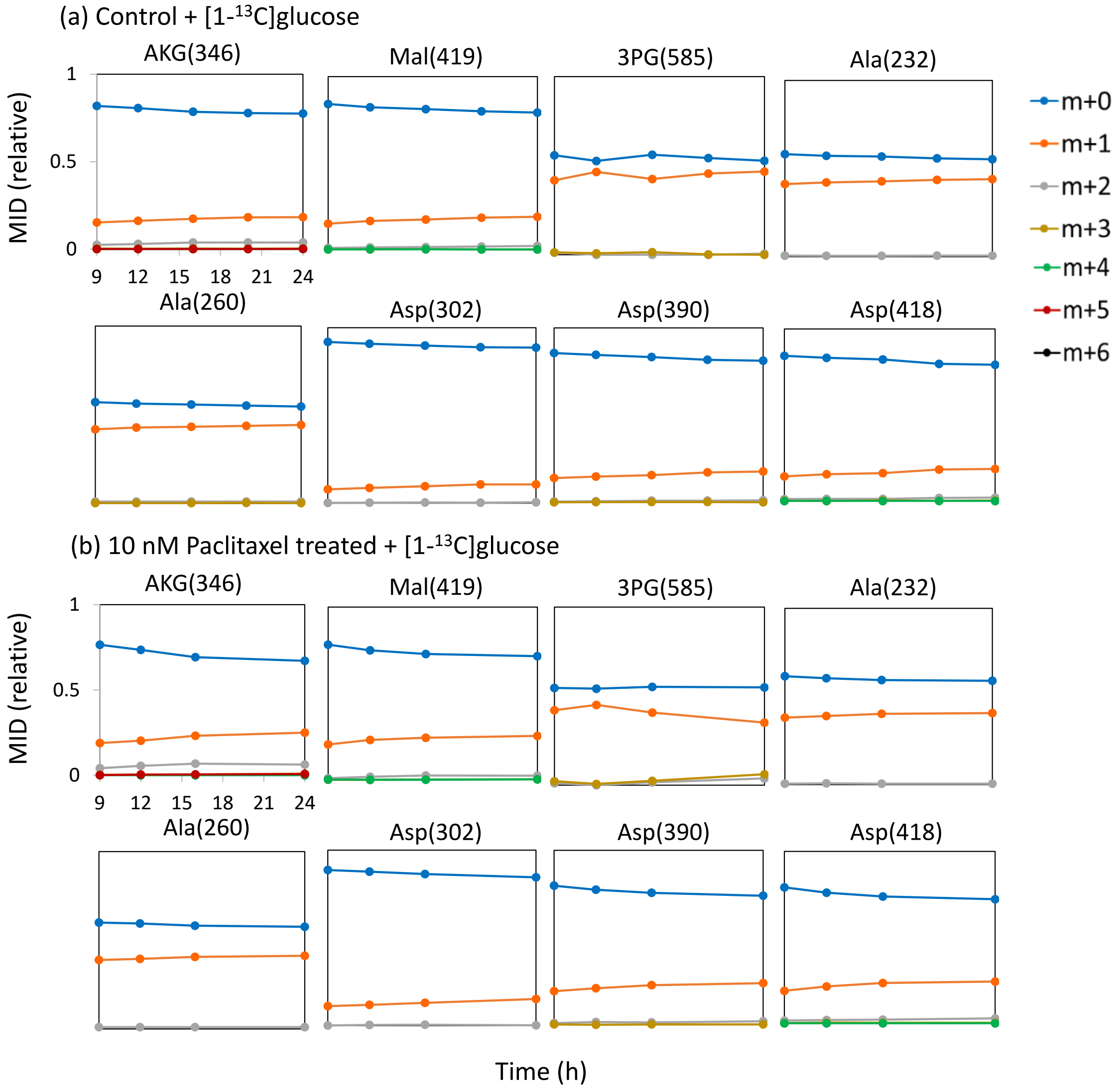

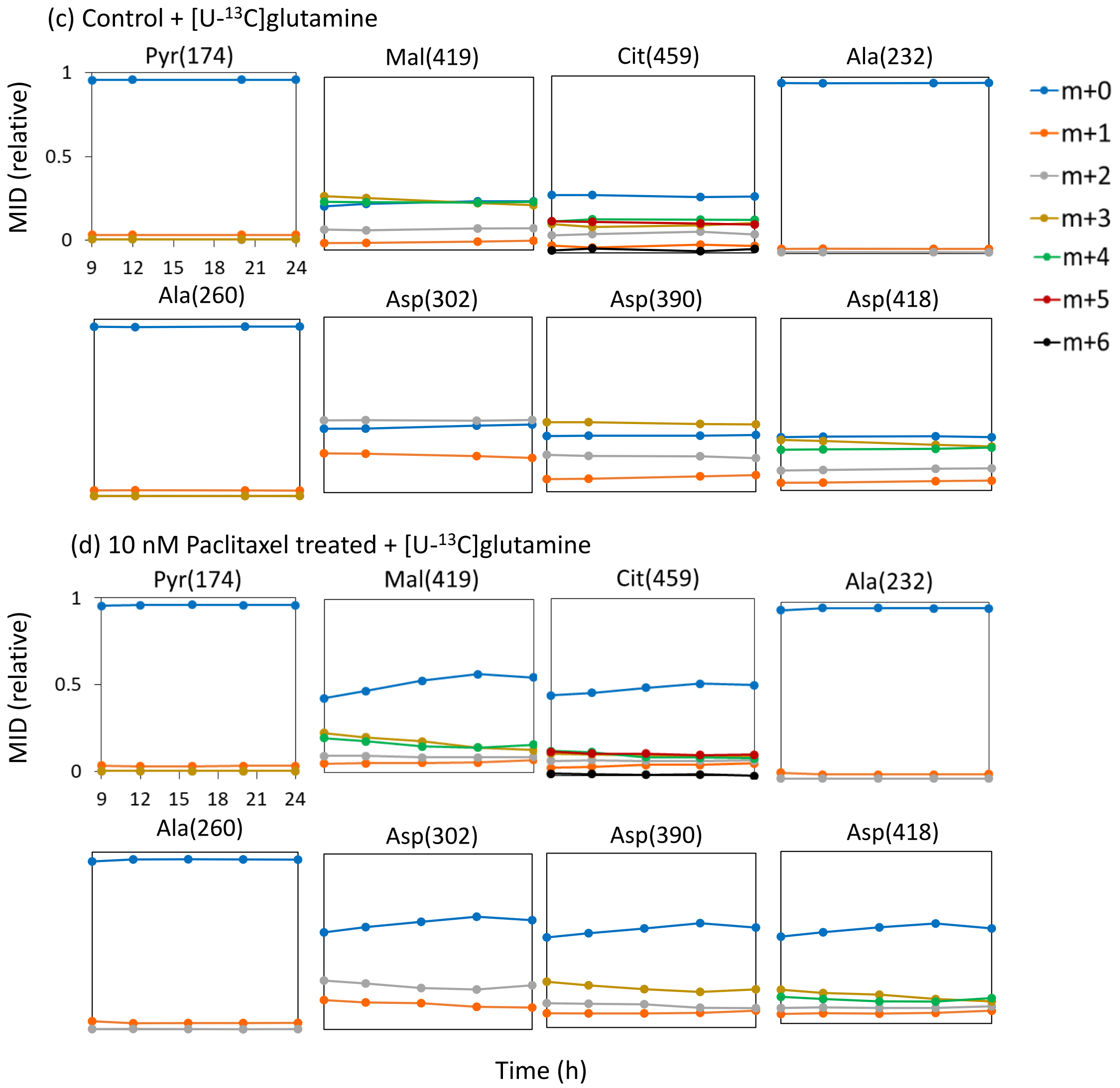

(c) Control + [U-13C]glutamine
MID (relative)
Pyr(174)
Mal(419)
Cit(459)
Ala(232)
Ala(260)
Asp(302)
Asp(390)
Asp(418)
1
0.5
0
9 12 15 18 21 24
m+0
m+1
m+2
m+3
m+4
m+5
m+6
(d) 10 nM Paclitaxel treated + [U-13C]glutamine
MID (relative)
Pyr(174)
Mal(419)
Cit(459)
Ala(232)
Ala(260)
Asp(302)
Asp(390)
Asp(418)
1
0.5
0
9 12 15 18 21 24
Time (h)
